# Supplementary material for: Hypothalamus-hippocampus circuitry regulates impulsivity via melanin-concentrating hormone
Source: Nat Commun. 2019 Oct 29;10:4923. doi: 10.1038/s41467-019-12895-y (PMC6820566; doi:10.1038/s41467-019-12895-y)
Supplement: Supplementary file 1 — Supplementary Information [file 41467_2019_12895_MOESM1_ESM.pdf]

Hypothalamus-hippocampus circuitry regulates impulsivity via melanin-concentrating hormone

Noble et al.

Supplementary methods

vHP projection-specific MCH knockdown

A custom short hairpin RNA (shRNA) targeting pMCH mRNA was cloned and packaged into an adeno-associated virus (AAV2[retro] under the control of a U6 promoter and co-expressing green fluorescent protein (GFP; AAV[retro]-GFP-U6-rPMCH-shRNA; Vector Biolabs, Malvern, PA, USA). For screening, first 4-5 shRNA candidates were transfected into HEK293 cells to compare the knockdown efficiency for each shRNA. A reporter assay was used to assess the knockdown efficiency of each shRNA candidates. The selected shRNA sequence was validated in vitro for 94% knockdown of the mRNA for PMCH. The sequence is as follows:

5'-CACC GCCTCTCTTCCTACATGTTAACTCGAGTTAACATGTAGGAAGAGAGGC-TTTTT-3'

The targeting sequence is GCCTCTCTTCCTACATGTTAA and the hairpin loop sequence is CTCGAG. This virus is now commercially available from Vector Biolabs (Malvern, PA, USA) upon request. An AAV2 (retro)-eSYN-EGFP virus from Vector Biolabs was used as a control virus. Animals were divided into similar groups matched on their average efficiency scores from their last 2 days of DRL 20 training. AAVs were delivered bilaterally to the vHP (AP: -4.9 ML: +/- 4.8 DV: -7.8, with DV zero at the skull at the injection site) at an injection volume of 200nl via pressure injection using the stereotaxic procedures described previously in *Methods*. DRL testing occurred following 3 weeks of incubation with the virus. Testing occurred thrice over three consecutive

days, then food was removed for 24 hours and animals were tested following 24 hours of food restriction for one additional test session. One week following DRL testing, Open Field activity testing was conducted as previously described in *Methods*.

Quantitative PCR was conducted to examine whether pMCH mRNA knockdown in vHP-projecting LHA neurons influenced global expression of MCH in the LHA. Brains from either control or pMCH shRNA AAV-injected animals were rapidly removed and flash frozen in isopentane and stored at -80$_{o}$C. Total RNA was extracted from 1mm micropunches (taken in cryostat) that targeted the MCH neuron-enriched LHA region (-2.6 mm posterior to bregma) according to manufacturer's instructions using RNeasy Lipid Tissue Mini Kit (Qiagen, Hilden, Germany). RNA was reverse transcribed to cDNA using the Quantitect Reverse Transcription Kit (Qiagen). qPCR was performed with TaqMan Universal PCR Master Mix (Applied Biosystems, Foster City, CA) using the BIO-RAD CFX96 Optics Model realtime PCR machine. Negative reverse-transcribed samples were generated, and all reactions were carried out in triplicate. The following TaqMan probes were used: *mch*: Rn00561766_g1, *Gapdh:* Rn01775763_g1. To determine relative expression values, the $2_{-\Delta\Delta CT}$ method was used, where triplicate Ct values for each sample were averaged and subtracted from those derived from GAPDH as previously described [1]. Results revealed that LHA MCH mRNA expression was reduced by ~7% in pMCH shRNA AAV-injected animals relative to control AAV-injected animals, an outcome consistent with our anatomical data estimating that between ~5-15% of all MCH neurons project to the vHP. However, this 7% reduction in MCH mRNA expression in the experimental group failed to reach statistical significance (means [SEM]: Control AAV 1.0 [0.139], pMCH shRNA AAV 0.889 [0.195].

For the 16 animals whose tissue underwent flash freezing and qPCR analyses, vHP AAV injections sites were confirmed by collecting 40μm sections and visualizing injection tracks and sites in darkfield microscopy, resulting in the removal of two animals from the pMCH shRNA group and one animal from the control AAV group from all analyses (based on lack of confirmation of injection track and site), leaving n=9 and n=8 for the experimental and control groups, respectively. The presence of GFP transgene expression at the vHP injection site and in retrogradely transfected LHA MCH neurons was confirmed in two additional animals from each group that were transcardially perfused for GFP immunohistochemistry.

Dual virus neuroanatomical tracing

To identify collateral projections from MCH neurons that project to the vHP, a dual virus neuroanatomical approach was used (n=3). A single pressure injection of 200 nl of the retrogradely transported AAV2 retro hSYN1-eGFP-2a-iCRE-wPRE (Vector Biolabs; 1.2 x 10^{13} GC/mL) was injected into the vHP using methods described previously in *Methods*. The injection coordinates were (AP: -4.9 ML: +/- 4.8 DV: -7.8). Four additional injections (200 nl each) of AAV1-CAG-FLEX-tdTomato-wPRE (Penn Vector Core, Philadelphia, PA; titer 4.26 x10^{13} GC/mL) were targeted to the lateral hypothalamus using the following coordinates: 1) -2.6 mm AP, ± 1.8 mm ML, -8.0 DV; 2) -2.6 mm AP, ± 1.0 mm ML, -8.0 DV; 3) -2.9 mm AP, ± 1.1 mm ML, -8.8 DV; 4) -2.9 mm AP, ± 1.6 mm ML, -8.8 DV (DV coordinates are relative to the skull surface at bregma). Animals were perfused 14 days after virus injections and immunofluorescence staining was performed for MCH as described previously in *Methods*. For the secondary antibody, Alexafluor 647 was used to avoid overlap with the AAV2 retro GFP virus and

pseudocolored green for the representative image. Photomicrographs were acquired using a Nikon 80i (Nikon DS-QI1,1280X1024 resolution, 1.45 megapixel) under epifluorescence.

Supplementary Table 1. Summary of functional brain mapping results

Brain regions	Left | Right hemisphere	
Cortical regions (cortical plate and subplate)		
ACAd/ACAv: anterior cingulate area, dorsal/ventral part	+	+
AId/AIv: agranular insular area, dorsal/ventral part		+
AIp: agranular insular area, posterior part	-	
AON: anterior olfactory nucleus		+
AUD: auditory area	+	+
BLA: basolateral amygdala nucleus	-*	-
BMA: basomedial amygdalar nucleus	-*	-
CA1: CA1 area, dorsal and ventral	+*	+
CA3: CA3 area, dorsal	+	
CLA: claustrum	+	
COA: cortical amygdalar nucleus	-*	-
ECT: ectorhinal area	+*	+*
ENT: entorhinal area	+	+
EPd: endopiriform nucleus, dorsal part		+*
DG: dentate gyrus	+	
GU: gustatory area	-*	
ILA: infralimbic area	+	+
LA: lateral amygdalar nucleus	-	
MOp/MOs: primary/secondary motor areas	-	
ORBv: orbital area, ventral part		+
PIR: piriform area, anterior aspect	+	+
PIR: piriform area, posterior aspect	-	
PL: prelimbic area	+	+
PTLp: parietal region, posterior association areas	+*	
RSPd/RSPv: retrosplenial area, dorsal/ventral part	+	+*
SSp: primary somatosensory area	-*	-*
SSs: supplemental somatosensory area	-	-
SUBv: subiculum, ventral part	+	+
TEa: temporal association areas	+*	+*
TT: tenia tecta		+
VISC: visceral area	-	-
VIS: visual areas	+*	+
Subcortical regions		
AAA: anterior amygdala area	-*	-
ACB: nucleus accumbens	+	+*
AHA: anterior hypothalamic area	-	
CEA: central nucleus amygdala	-*	-*
CENT: cerebellar central lobule	+	+

CLI: central linear nucleus raphe	-	-
CP: caudoputamen	-*	-*
CSm/CSl: superior central nucleus raphe, medial/lateral part	+	+
DMH: dorsomedial hypothalamic nucleus	-	-
FS: striatal fundus	-*	
ICe: inferior colliculus, external nucleus	-*	
III: oculomotor nucleus		-
IPN: interpeduncular nucleus	+*	+*
LD: lateral dorsal nucleus thalamus	+*	
LGd: lateral geniculate complex, dorsal part	+*	+
LHA: lateral hypothalamic area	-*	-*
LS: lateral septal nucleus	-	-
MD: mediodorsal nucleus thalamus	-	-
MEA: medial amygdalar nucleus	-*	-
OT: olfactory tubercle	-*	
PAGd: periaqueductal gray (dorsal division)	+	
PB: parabrachial nucleus		+
POR: periolivary nuclei		+
RH: rhomboid nucleus	+*	
RL: rostral linear nucleus raphe	-	-*
RN: red nucleus		-
RR: midbrain reticular nucleus, retrorubral area		-*
RT: reticular nucleus thalamus	+	+*
SC: superior colliculus	+*	+
VAL: ventral anterior-lateral complex thalamus	-	
VM: ventral medial nucleus thalamus	-	
VPL: ventral posterolateral nucleus thalamus		+*
VTA: ventral tegmental area	-	-
ZI: zona incerta	+	

Statistically significant increases or decreases in regional cerebral 2-deoxy-D-glucose uptake in melanin-concentrating hormone compared to vehicle treated group are noted with + or -, respectively (Student's *t*-test, voxel level $P < 0.05$ for clusters > 200 contiguous voxels). *: significance is shown at the voxel level $P < 0.01$ for clusters > 200 contiguous voxels. Cortical regions include cortical plate and subplate as defined by Swanson (2004), from which abbreviations are taken with minor modifications.

Supplementary Table 2. MCH-related changes in functional connectivity of the ventral CA1

	MCH group		Control group	
	r	*P*	*r*	*P*
Cortical regions (cortical plate and subplate)				
ACAd/ACAv: anterior cingulate area, dorsal/ventral	- 0.89*	0.001	+0.27	0.5
AId/AIv: agranular insular area, dorsal/ventral part	- 0.77*	0.02	+0.22	0.6
AIp: agranular insular area, posterior part	- 0.75	0.02	- 0.39	0.3
AON: anterior olfactory nucleus	+0.84	0.005	+0.35	0.4
CA1: CA1 area, dorsal	+0.85*	0.004	+0.04	0.9
CA3: CA3 area, dorsal	+0.79	0.01	+0.47	0.2
DG: dentate gyrus	+0.74	0.02	+0.64	0.09
ECT: ectorhinal area	+0.80	0.01	+0.21	0.6
ENTl: entorhinal area, lateral part	+0.94*	0.0001	+0.38	0.4
MOp/MOs: primary/secondary motor areas	- 0.90*	0.001	- 0.11	0.8
PIR: piriform area, anterior aspect	- 0.84	0.004	- 0.68	0.06
RSPd/RSPv: retrosplenial area, dorsal/ventral part	- 0.86*	0.003	- 0.07	0.9
SSs: supplemental somatosensory area	- 0.84	0.004	- 0.46	0.2
SUBv: subiculum, ventral part	+0.78	0.01	+0.22	0.6
TEa: temporal association areas	+0.75	0.02	+0.35	0.4
TR: postpiriform transition area	+0.84	0.005	+0.11	0.8
VISC: visceral area	- 0.92	0.0005	- 0.89	0.004
Subcortical regions				
ACB: nucleus accumbens	+0.76	0.02	+0.09	0.8
AD/AV: anterodorsal/anteroventral nucleus thalamus	- 0.90*	0.0009	- 0.12	0.8
CENT: cerebellar central lobule	+0.91*	0.0008	- 0.65	0.08
CM: central medial nucleus thalamus	+0.84*	0.005	- 0.80	0.02
CP: caudoputamen	- 0.91*	0.0007	+0.29	0.5
DR: dorsal nucleus raphe	+0.75*	0.02	- 0.26	0.5
ICe: inferior colliculus, external nucleus	+0.94*	0.0002	- 0.16	0.7
LGd: lateral geniculate complex, dorsal part	+0.92	0.0005	+0.50	0.2
LH: lateral habenula	- 0.88*	0.002	+0.27	0.5
MD: mediodorsal nucleus thalamus	- 0.84	0.004	- 0.17	0.7
PAG: periaqueductal gray	+0.81*	0.008	- 0.26	0.5
PB: parabrachial nucleus	+0.75	0.02	+0.21	0.6
PRNc: pontine reticular nucleus, caudal part	+0.85	0.004	+0.11	0.8
PRNr: pontine reticular nucleus, rostral part	+0.93*	0.0003	+0.10	0.8
RE: nucleus reuniens, thalamus	- 0.80*	0.01	+0.56	0.2
SC: superior colliculus	+0.76	0.02	- 0.19	0.7
VAL: ventral anterior-lateral complex thalamus	- 0.74	0.02	- 0.23	0.6
VM: ventral medial nucleus thalamus	- 0.76*	0.02	+0.48	0.2
VPL: ventral posterolateral nucleus thalamus	+0.86*	0.003	- 0.43	0.3
ZI: zona incerta	+0.94*	0.0002	- 0.43	0.3

Pearson's correlation coefficient (*r*) and *P* value are listed for regions showing significant correlations with the ventral CA1 seed in regional cerebral 2-deoxy-D-glucose uptake in the melanin-concentrating hormone (MCH) group. Focus is put on findings in the left hemisphere

where the seed was defined. Cortical regions include cortical plate and subplate as defined by Swanson (2004), from which abbreviations are taken with minor modifications. *: statistically significant between-group difference in correlation coefficient (Fisher's Z-transform, $P < 0.05$).

Supplementary Figure 1

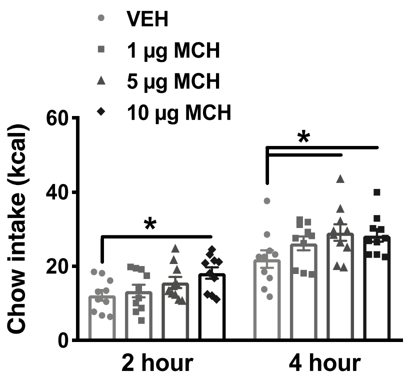

Supplementary Figure 1: Intracerebroventricular MCH increases chow intake Effect of intracerebroventricular (i.c.v.) MCH injection on standard chow intake in the home cage (n=10). Doses of 1, 5, and 10 µg of MCH were injected using a counterbalanced, within subjects' design. Results revealed a main effect of time ($F_{(2, 72)}$ = 188.4; P<.0001) and drug ($F_{(3, 36)}$ = 4.03; P=.014). Data were analyzed using a repeated measures ANOVA with Dunnett's multiple comparison test for post hoc analyses (*P<.05). Data shown as mean ± SEM. Source data are provided as a source data file.

Supplementary Figure 2

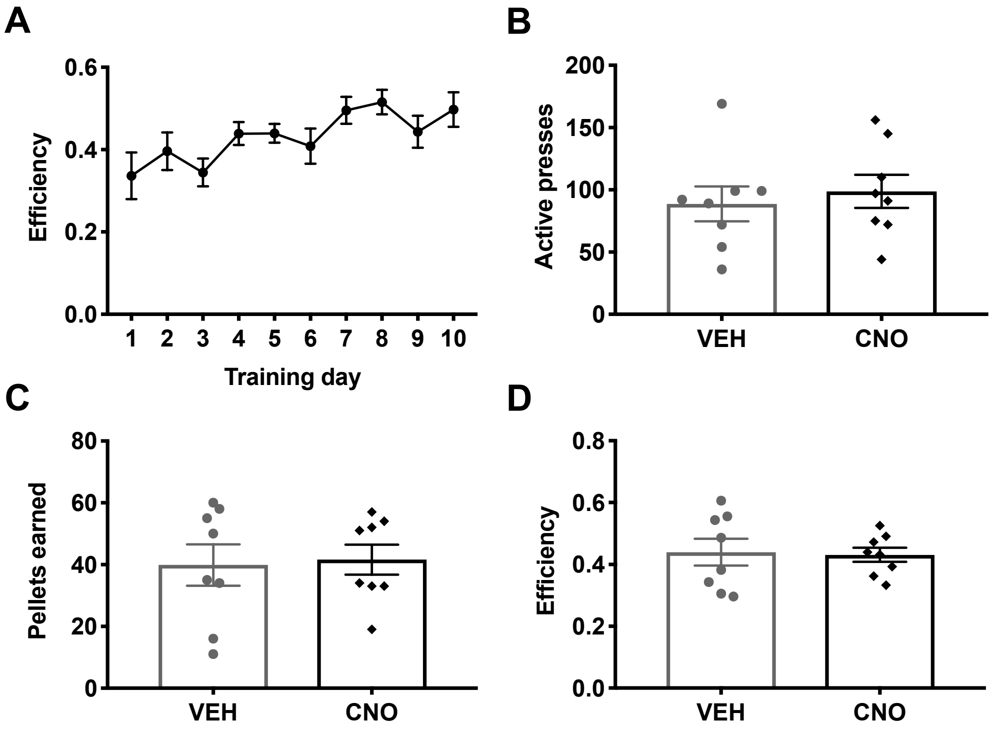

Supplementary Figure 2: Clozapine-N-oxide (CNO) without DREADDs

Effect of i.c.v. injection of CNO in animals not injected with the DREADDs virus on performance in the differential reinforcement of low rates of responding (DRL) task (n=8). Animals were injected using a counterbalanced, within-subjects design: (A) efficiency data during the training phase in DRL 20. (B) number of active lever presses during test phase. (C) number of pellets earned during test phase. (D) efficiency in the DRL test phase. Data were analyzed using a Student's two-tailed paired t-test. Data shown as mean ± SEM. Source data are provided as a source data file.

Supplementary Figure 3

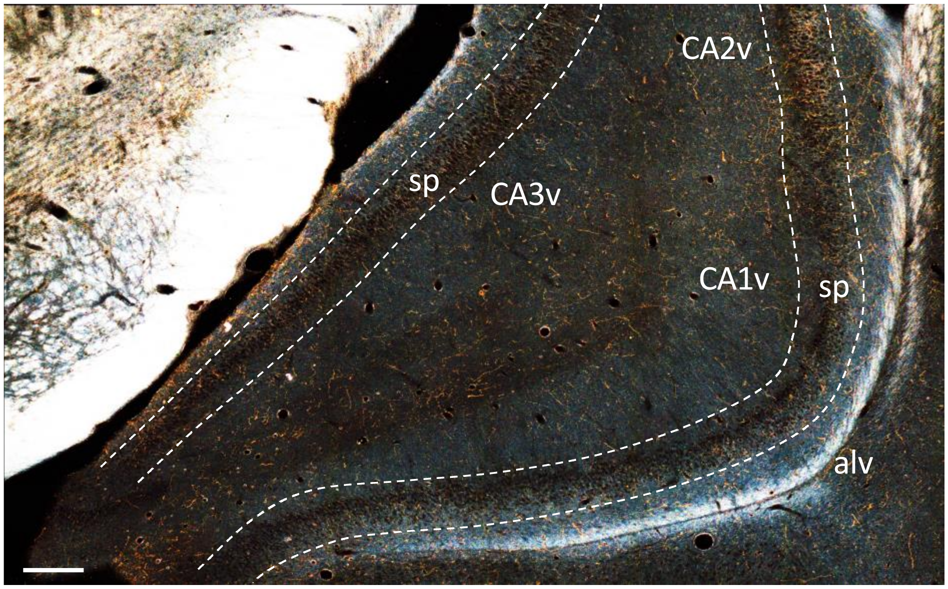

Supplementary Figure 3: The vHP contains MCH immunoreactive axons

A representative coronal image containing immunohistochemical staining for MCH (orange fibers) against a darkfield image of the vHP taken from a 30 µm section cut 4.6 mm caudal to bregma. Abbreviations: alv = Alveus; CA1v= field CA1 of the ventral hippocampus; CA2s= field CA2 of the ventral hippocampus; CA3v= field CA3 of the ventral hippocampus; sp = Pyramidal layer; scale bar = 200 µm

Supplementary Figure 4

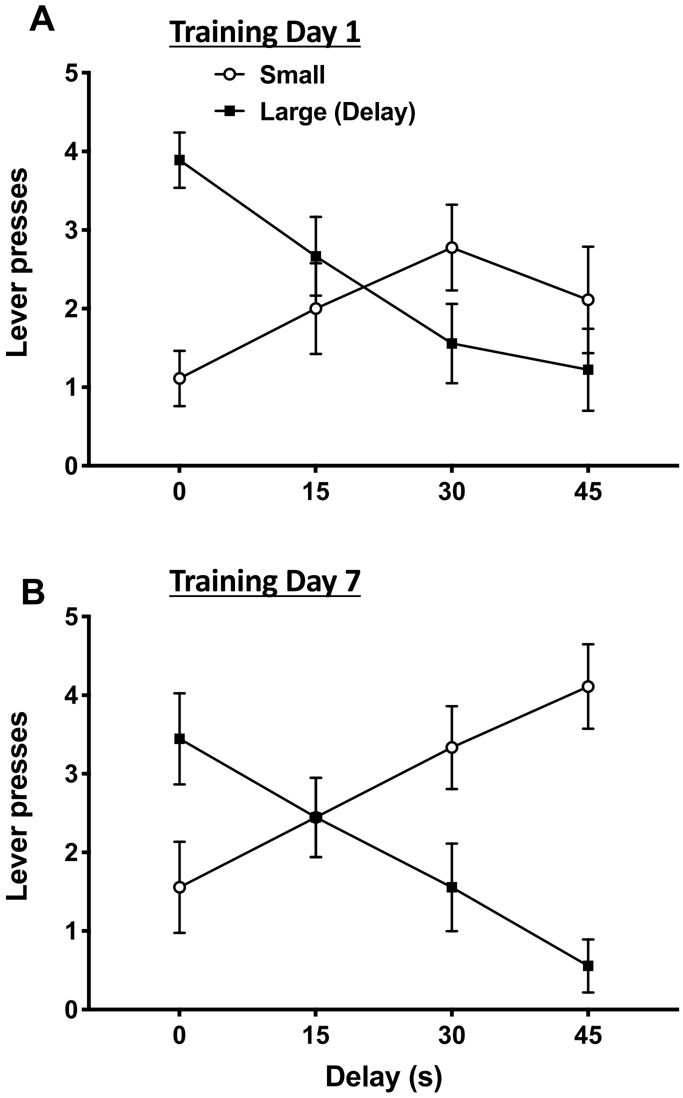

Supplementary Figure 4: Delay Discounting training data After 7 days of training in the delay discounting task, animals respond more on the lever that delivers the immediate reward (small, 1 pellet delivered immediately post lever press) compared with the lever that delivers the larger reward after an incremental delay large (delay) of 0, 15, 30, or 45 seconds (s). Source data are provided as a source data file.

Supplementary Figure 5

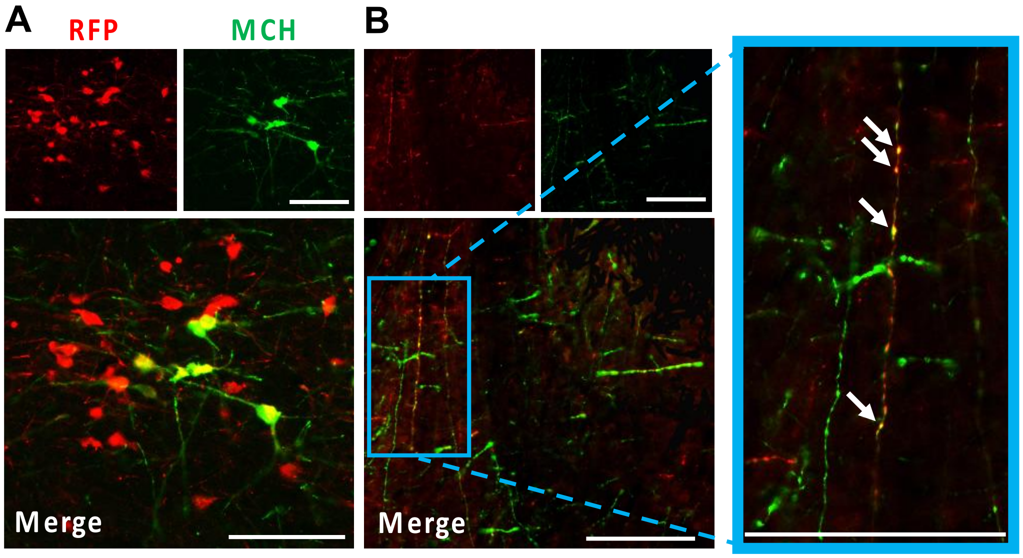

Supplementary Figure 5: Collateral projections of vHP-projecting MCH neurons in the basolateral amygdala Representative images showing (A) tdTomato immunoreactive cell bodies (cells that project to the vHP; red) colocalized with MCH immunoreactive cell bodies (green) in the lateral hypothalamus, and (B) colocalization of MCH and tdTomato immunoreactive axons in the basolateral amygdala. White arrows point to areas of MCH and mCherry axonal colocalization; scale bars = 50 μm.

Supplementary Figure 6

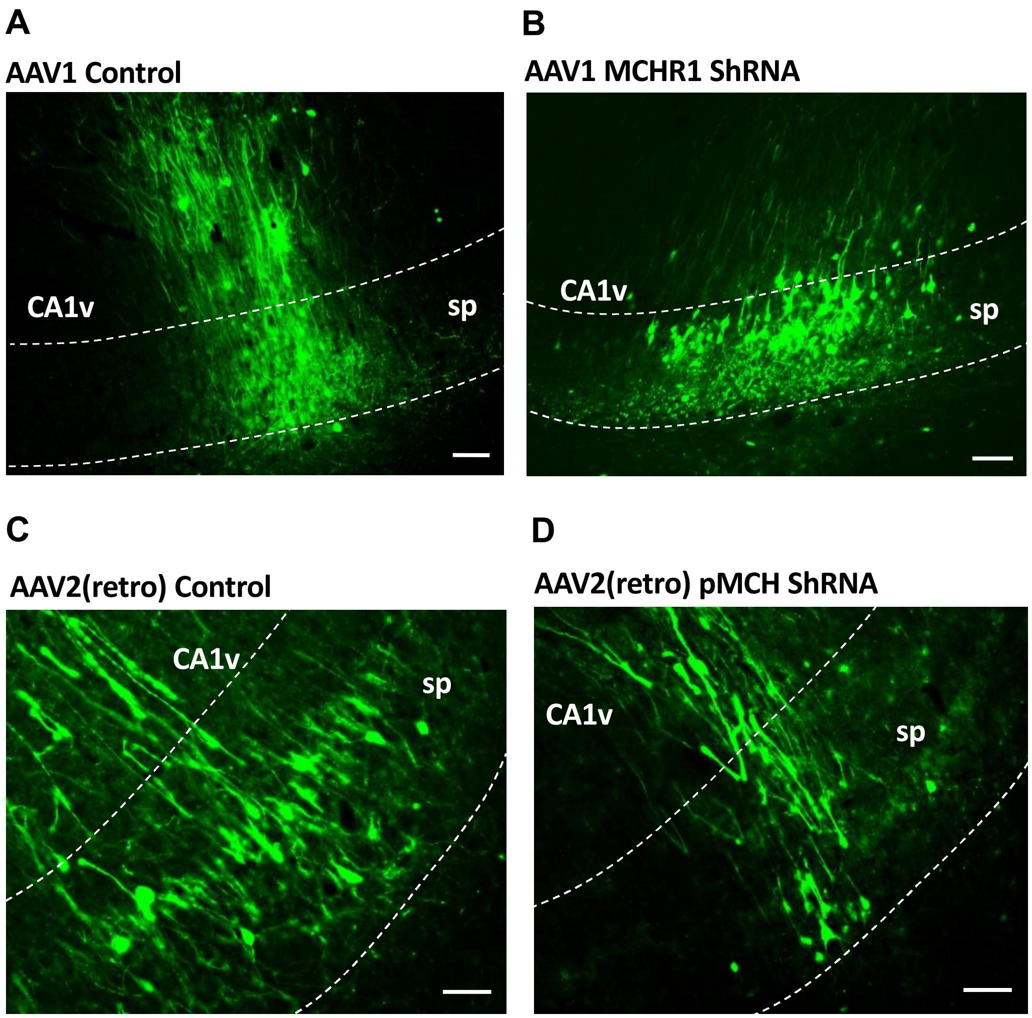

Supplementary Figure 6: Injection sites for control and shRNA viruses

(A) Representative injection sites for the AAV-scrambled-shRNA-GFP control virus (Control), (B) AAV-MCHR1-shRNA-GFP virus (MCHR1 ShRNA), (C) AAV2 (retro)-eSYN-EGFP, and (D) AAV[retro]-GFP-U6-rPMCH-shRNA in the CA1 subregion of the vHP. Images are from a 30 µm coronal section taken from a rat brain at 4.7 mm posterior to bregma. Images were taken at 20x magnification. Abbreviations: CA1v= field CA1 of the ventral hippocampus; sp = Pyramidal layer; scale bar=50µm.

Supplementary Figure 7

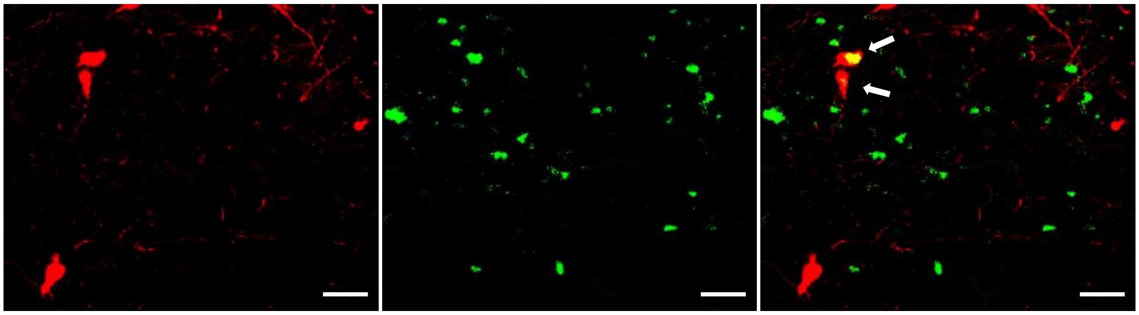

Supplementary Figure 7: MCH shRNA reporter colocalization with MCH neurons Representative images showing MCH (red), GFP (green), and colocalization (yellow) in the lateral hypothalamus of a representative that received injections of AAV[retro]-GFP-U6-rPMCH-shRNA in the CA1 subregion of the vHP. Images are from a 30 μm coronal section taken from a rat brain at 2.7 mm posterior to bregma. Images were taken at 20x magnification; scale bar=50μm.

Supplementary Figure 8

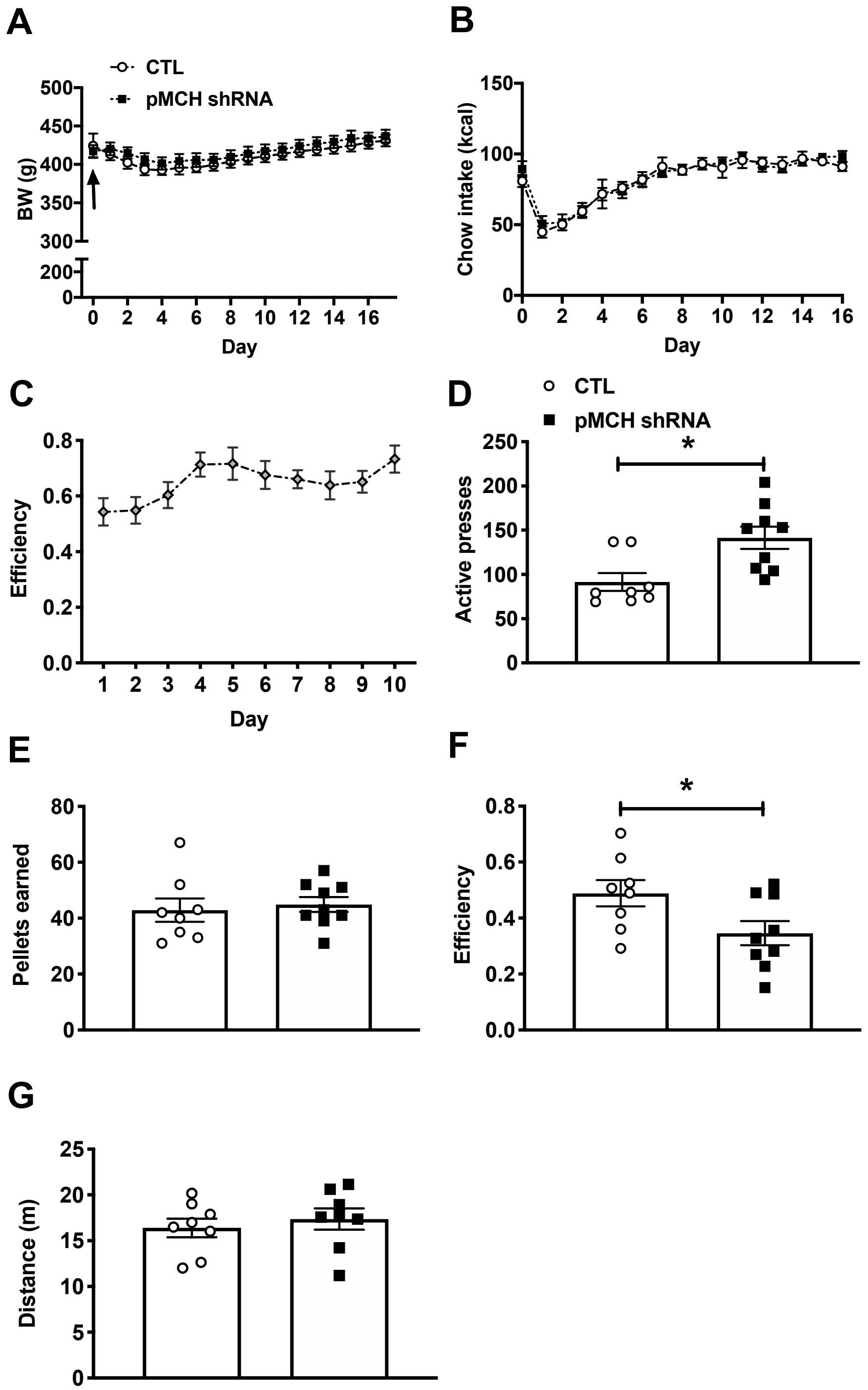

Supplementary Figure 8: Effects of MCH shRNA targeting vHP-projecting MCH neurons (A) Body weight and (B) chow intake before (day 0) and after surgery in animals injected with either control virus (AAV2 [retro]-GFP [CTL]; n=8) or AAV2

(retro) pMCH shRNA (pMCH shRNA); n=9; arrow in A indicates surgery date). (C) Efficiency data during the training phase in DRL 20 (before AAV injections). (D-H; student's two-tailed, unpaired t-test; n=8, 9) DRL scores in animals following 24 hour food restriction when tested following 3-week RNAi AAV incubation period: (D) Number of active lever presses, (E) number of pellets earned, (F) efficiency, and (G) lever presses on the inactive lever. (H) Distance travelled in the open field task following 3-week RNAi AAV incubation period. Data shown as mean ± SEM (*$P<0.05$). Source data are provided as a source data file.

References

1. Davis EA, Zhou W, Dailey MJ. Evidence for a direct effect of the autonomic nervous system on intestinal epithelial stem cell proliferation. *Physiological reports* **6**, e13745 (2018).
