# Supplementary material for: Hypothalamus-hippocampus circuitry regulates impulsivity via melanin-concentrating hormone
Source: Nat Commun. 2019 Oct 29;10:4923. doi: 10.1038/s41467-019-12895-y (PMC6820566; doi:10.1038/s41467-019-12895-y)
Supplement: Supplementary file 3 — Reporting Summary [file 41467_2019_12895_MOESM3_ESM.pdf]

nature research

Corresponding author(s): Scott E Kanoski, PhD

Last updated by author(s): Oct 8, 2019

Reporting Summary

Nature Research wishes to improve the reproducibility of the work that we publish. This form provides structure for consistency and transparency in reporting. For further information on Nature Research policies, see Authors & Referees and the Editorial Policy Checklist.

Statistics

For all statistical analyses, confirm that the following items are present in the figure legend, table legend, main text, or Methods section.

n/a	Confirmed	
☐	☒	The exact sample size (*n*) for each experimental group/condition, given as a discrete number and unit of measurement
☐	☒	A statement on whether measurements were taken from distinct samples or whether the same sample was measured repeatedly
☐	☒	The statistical test(s) used AND whether they are one- or two-sided<br>*Only common tests should be described solely by name; describe more complex techniques in the Methods section.*
☒	☐	A description of all covariates tested
☐	☒	A description of any assumptions or corrections, such as tests of normality and adjustment for multiple comparisons
☐	☒	A full description of the statistical parameters including central tendency (e.g. means) or other basic estimates (e.g. regression coefficient) AND variation (e.g. standard deviation) or associated estimates of uncertainty (e.g. confidence intervals)
☐	☒	For null hypothesis testing, the test statistic (e.g. *F*, *t*, *r*) with confidence intervals, effect sizes, degrees of freedom and *P* value noted<br>*Give P values as exact values whenever suitable.*
☒	☐	For Bayesian analysis, information on the choice of priors and Markov chain Monte Carlo settings
☒	☐	For hierarchical and complex designs, identification of the appropriate level for tests and full reporting of outcomes
☒	☐	Estimates of effect sizes (e.g. Cohen's *d*, Pearson's *r*), indicating how they were calculated

Our web collection on statistics for biologists contains articles on many of the points above.

Software and code

Policy information about availability of computer code

Data collection	Custom code was created for data collection using Med- Associates software (Med Associates, Fairfax, VT, USA). This code is available upon request. TurboReg, an automated pixel-based registration algorithm, was implemented in ImageJ (version 1.35, http://rsbweb.nih.gov/ij/). The statistical parametric mapping (SPM) package (Wellcome Centre for Neuroimaging, University College London, London, UK) was used for the analysis of rodent autoradiographic cerebral blood flow. Brains were spatially normalized in SPM (version 5). The structural ROI of the ventral CA1 was hand drawn in MRIcro (version 1.40, http://cnl.web.arizona.edu/mricro.htm) over the template brain in the left hemisphere according the rat brain atlas. Mean optical density of the seed ROI was extracted for each animal using the MarsBaR toolbox for SPM (version 0.42, http://marsbar.sourceforge.net/).
Data analysis	Statistics were performed using GraphPad Prism 7.0 Software (GraphPad Software Inc., San Diego, CA, USA), Microsoft Excel for Mac (v. 15.26; Microsoft Inc., Redmond, WA, USA), or SPM (version 0.42, http://marsbar.sourceforge.net/) (detailed in the manuscript).

For manuscripts utilizing custom algorithms or software that are central to the research but not yet described in published literature, software must be made available to editors/reviewers. We strongly encourage code deposition in a community repository (e.g. GitHub). See the Nature Research guidelines for submitting code & software for further information.

Data

Policy information about availability of data

All manuscripts must include a data availability statement. This statement should provide the following information, where applicable:

- Accession codes, unique identifiers, or web links for publicly available datasets
- A list of figures that have associated raw data
- A description of any restrictions on data availability

All data generated and analyzed for this manuscript are available from the corresponding author (S.E.K.) upon reasonable request. The source data underlying Figs. 1B-E, 1G-J, 3A-D, 3G-J, 4A-I, 5B-H, Supplemental Fig 1, Supplemental Fig 2A-D, Supplemental Fig 4A,B, Supplemental Fig 7A-G, and all inactive lever press data as well as the qPCR data for the pMCH RNAi experiment are provided as a Source Data file. The data from this manuscript are available in the Open Science Framework Repository

[https://doi.org/10.17605/OSF.IO/4XEVQ].

Field-specific reporting

Please select the one below that is the best fit for your research. If you are not sure, read the appropriate sections before making your selection.

☒ Life sciences ☐ Behavioural & social sciences ☐ Ecological, evolutionary & environmental sciences

For a reference copy of the document with all sections, see nature.com/documents/nr-reporting-summary-flat.pdf

Life sciences study design

All studies must disclose on these points even when the disclosure is negative.

Sample size	The number of animals to be used for each proposed experiment is based on power analyses conducted that considered our own pilot work. Power analyses were conducted for each proposed experiment using Statsoft software (Statistica, V7). Alpha level was set at 0.05 for power analyses; the Root Mean Square Standardized Effect (RMSSE) was estimated separately for each experiment based on estimated means and standard deviations from our pilot data. The group sizes were chosen based on the minimum number of animals to provide statistical power to achieve detection of statistically significant differences based on the power analyses described above.
Data exclusions	Animals were excluded based on the following pre-determined criteria: 1) where they failed learn to lever press during the training phase of the DRL task (n=4 for all experiments) 2) where they failed to meet the indicated (in the manuscript) minimum criterion of mCherry positive cells in the DREADDs experiments (n= 1) where they were determined to be statistically significant outliers based on the Grubb's test using an alpha level of .05 (n=4 for all experiments) .
Replication	All attempts to replicate experiments were successful.
Randomization	Though most of the experiments utilized a within-subjects design, the order of treatments was counterbalanced based on matching for initial body weight where lever pressing data was not relevant to the experiment, or based on matching for lever pressing activity prior to test day for tasks in the operant chambers. For shRNA experiment, animals were randomized such that there were no differences in body weight or efficiency in DRL training prior to surgery.
Blinding	Investigators running behavioral experiments, and weighing food intake and body weights were blinded to treatment groups during data collection.

Reporting for specific materials, systems and methods

We require information from authors about some types of materials, experimental systems and methods used in many studies. Here, indicate whether each material, system or method listed is relevant to your study. If you are not sure if a list item applies to your research, read the appropriate section before selecting a response.

Materials & experimental systems

n/a	Involved in the study	
☐	☒	Antibodies
☒	☐	Eukaryotic cell lines
☒	☐	Palaeontology
☐	☒	Animals and other organisms
☒	☐	Human research participants
☒	☐	Clinical data

Methods

n/a	Involved in the study	
☒	☐	ChIP-seq
☒	☐	Flow cytometry
☒	☐	MRI-based neuroimaging

Antibodies

Antibodies used	The following antibodies and dilutions were used: rabbit anti-MCH (1:1000 or 1:2000 where noted; Phoenix Pharmaceuticals H-070-47 lot # 01629-8, Burlingame, CA, USA), rabbit anti-RFP (1:2000, Rockland Inc. #600-401-379 lot #35868, Limerick, PA, USA), Guinea Pig anti fluorogold (1:5000; Protos Biotech Corp., New York, NY, USA). All secondary antibodies were obtained from Jackson Immunoresearch and used at 1:500 dilution (Jackson Immunoresearch; West Grove, PA, USA).
Validation	The MCH primary was validated based on comparisons for MCH positive signal colocalized with mCherry immunofluorescence in animals expressing the MCH-mCherry transgene (delivered via virogenetics). RFP and Fluorogold antibodies showed no immunoreactivity in non-injected animals, confirming the specificity of RFP and Fluorogold to either fluorogold (injected) or mCherry (virogenetically delivered to MCH neurons).

Animals and other organisms

Policy information about studies involving animals; ARRIVE guidelines recommended for reporting animal research

Laboratory animals	Male Sprague Dawley rats (Envigo, Indianapolis, IN, USA) weighing 300-400g were used for this study.
Wild animals	N/A
Field-collected samples	N/A
Ethics oversight	Experiments were performed in accordance with NIH Guidelines for the Care and Use of Laboratory Animals, and all procedures were approved by the Institutional Animal Care and Use Committee of the University of Southern California.

Note that full information on the approval of the study protocol must also be provided in the manuscript.
